# Unique provision of allied health services to remote renal patient care

**DOI:** 10.1186/1757-1146-4-S1-P15

**Published:** 2011-05-20

**Authors:** Brydie M Donnelly, Sarah Reilly

**Affiliations:** 1 Boab Health Services, Broome, Western Australia 6725, Australia

## 

End stage renal failure (ESRF) occurs at a far greater rate in the Kimberley than the national average, almost trebling in the past ten years. Many people affected are Indigenous Australians. The Kimberley Satellite Dialysis Centre (KSDC), the first of its kind in Australia, is managed by an Aboriginal Community Controlled Health Service (ACCHS). Operating since October 2002, it is located over 2 000 kilometers from the nearest tertiary support centre. Ten haemodialysis machines and beds, cater for 42 clients with ESRF, and provide respite care to patients undergoing home haemodialysis, to avoid the long journey to Perth. Staff include Registered Nurses (RN’s), Aboriginal Health Workers (AHW’s), administrative workers, a social worker, medical officer, and drivers to assist in transportation. Many of these employees are Aboriginal people, and therefore improve the cultural appropriateness of the service provided. The Dieticians Association of Australia (DAA) recommend the gold standard of care for patients with chronic kidney disease include the services of a dietician. Renal-based nutrition advice was originally provided in 2006 for dialysis patients, however, in 2009 services were increased and now incorporate regular clinical dietetic services. Clients receive thorough assessments to identify and manage malnutrition, uncontrolled co-morbidities such as diabetes, mineral and bone disorders and electrolyte disturbances. Chronic kidney disease also increases the risk of developing foot complications, including ulceration and amputation. Risk factors are often exacerbated by a concomitant diagnosis of diabetes mellitus, and increase with progression of kidney disease. In 2006 a podiatry service began providing a monthly service to all patients undergoing treatment at the centre. Podiatric care in this setting focuses on assessment of risk level, patient education to promote healthy foot care practices, routine nail and skin care to prevent tissue damage, and wound care treatment, assessment and advice, where required. The podiatrist and the dietician work closely to ensure optimal care for patients, for instance those with chronic wounds. Multiple impediments to wound healing may exist, including inadequate nutrition and biomechanical abnormality, as well as working together on weight loss regimes for patients, enhancing mobility and potential for exercise, and providing dietary advice to reduce weight. Both clinicians work closely with the AHW’s RN’s, Physician and social worker to ensure continuation of care and adequate support is provided to the patient, depending on their particular need.

